# Comparative genomic analysis reveals bilateral breast cancers are genetically independent

**DOI:** 10.18632/oncotarget.5569

**Published:** 2015-09-10

**Authors:** Fangfang Song, Xiangchun Li, Fengju Song, Yanrui Zhao, Haixin Li, Hong Zheng, Zhibo Gao, Jun Wang, Wei Zhang, Kexin Chen

**Affiliations:** ^1^ Department of Epidemiology and Biostatistics, Tianjin Medical University Cancer Institute and Hospital, National Clinical Research Center of Cancer, Key Laboratory of Cancer Prevention and Therapy, Tianjin, P.R. China; ^2^ BGI-Shenzhen, Shenzhen, China; ^3^ Department of Medicine and Therapeutics, State Key Laboratory of Digestive Disease, Li Ka Shing Institute of Health Sciences, The Chinese University of Hong Kong, Hong Kong; ^4^ Department of Pathology, University of Texas MD Anderson Cancer Center, Houston, Texas, USA

**Keywords:** bilateral breast cancer, exome sequencing, array comparative genomic hybridization, clonality, genetic concordance

## Abstract

Bilateral breast cancer (BBC) poses a major challenge for oncologists because of the cryptic relationship between the two lesions. The purpose of this study was to determine the origin of the contralateral breast cancer (either dependent or independent of the index tumor). Here, we used ultra-deep whole-exome sequencing and array comparative genomic hybridization (aCGH) to study four paired samples of BBCs with different tumor subtypes and time intervals between the developments of each tumor. We used two paired primary breast tumors and corresponding metastatic liver lesions as the control. We tested the origin independent nature of BBC in three ways: mutational concordance, mutational signature clustering, and clonality analysis using copy number profiles. We found that the paired BBC samples had near-zero concordant mutation rates, which were much lower than those of the paired primary/metastasis samples. The results of a mutational signature analysis also suggested that BBCs are independent of one another. A clonality analysis using aCGH data further revealed that paired BBC samples was clonally independent, in contrast to clonal related origin found for paired primary/metastasis samples. Our preliminary findings show that BBCs in Han Chinese women are origin independent and thus should be treated separately.

## INTRODUCTION

Most cases of the breast cancer are unilateral, and bilateral breast cancer (BBC) occurs in approximately 5% of female breast cancer survivors [1, 2]; it is classified as either synchronous or metachronous on the basis of the time interval between the first and second contralateral tumors (generally 6 months) [3]. In clinical practice, approximately 1% of all breast cancers are synchronous and 3%-7% are metachronous [4]. BBC has been considered to possess similar phenotypic features to those of unilateral breast cancer, even though it is more commonly associated with a positive family history of breast cancer, early disease onset, lobular histologic type multicentricity of the first tumor, and BRCA1 and BRCA2 mutations [5-7]. The increasing breast cancer incidence, more favorable prognoses, extended life expectancies, and improvements in detection are expected to lead to an increased survival rate of BBC [8]. Thus, BBC holds intriguing clinical and fundamental aspects in understanding tumor-host relationships, considering the few paired organs in human body with both high cancer incidence and good cancer survival.

Nevertheless, BBC remains less studied than its unilateral counterpart and has posed a great challenge for oncologists due to many unanswered questions. First, the biological relationship between the two breast cancers is not well understood. It can be difficult to discriminate between a second primary tumor and a breast metastasis [9]. Clarifying the issue of BBC clonality has implications for our understanding of breast carcinogenesis and for identifying the optimal breast cancer treatment [5, 6], as the management of primary disease and recurrent or metastatic breast cancer is substantially different [7]. A misdiagnosis of BBC or metastasis may result in different therapeutic regimens and poor disease outcome. BBC clonality has been studied using X chromosome inactivation, p53 mutation detection, and DNA allelotyping [10-15]. The results of these conventional cytogenetic investigations indicate that the vast majority of clinically diagnosed BBCs, if not all, represent clonally independent disease. A few studies have used comparative genomic hybridization (CGH) to determine the clonal association of BBCs [16-18] but reported conflicting results. These were insufficient for drawing a conclusion about the clonal origin of BBC.

Second, although BBCs are subjected to the same environmental and genetic influences, there may be a complex combination of different time spans (synchronous or metachronous), morphologic characteristics, and hormonal receptor statuses [19]. Whether the dominant tumor hypothesis in molecular-genetic profiles stands is debatable [20, 21]. The currently available genetic evidence for breast cancer metastasis suggests that the time interval between the development of the two tumors is important in discriminating between true BBCs and contralateral metastasis [4]. Mostly synchronous metastases present with identical genetic patterns to those of the primary breast tumor. In the case of metachronous metastasis, additional genetic events also drive tumor evolution except for a common load of abnormalities to the primary lesions [4, 14, 22]. Nevertheless, these discordant mutations do not obscure the relationship between the two tumors. Thus, synchronous and metachronous BBC with highly concordant genetic profiles may correspond to contralateral metastasis. However, the results of comparative analyses of the concordance of tumor molecular expression characteristics and genetic patterns in synchronous or metachronous BBC were inconsistent and inconclusive [14, 18, 23, 24]. Further evidence from whole-genome investigations is needed to identify molecular-genetic profiles in synchronous and metachronous BBCs and address whether there is a tendency to concordance of genetic lesions in BBC.

In this study, we analyzed genomic alterations in four BBC tumor pairs and two primary-metastasis tumor pairs from patients with breast cancer and liver metastasis to shed light on the controversial issues of the clonal origins in BBC pathogenesis. With the use of whole-exome high-throughput sequencing and comprehensive array CGH (aCGH), we confirmed that BBC consists of two clonally independent malignancies. Interestingly, neither synchronous nor metachronous BBCs exhibited a tendency towards concordance of genetic routes.

## RESULTS

We examined the genomic landscape of BBCs by ultra-deep whole-exome sequencing of the bilateral tumors and matched blood samples. We selected four pairs of BBC samples for sequencing: synchronous 1-1: luminal, 1-2: luminal; synchronous 2-1: Her2-enriched, 2-2: luminal; metachronous 3-1: basal-like, 3-2: basal-like; and metachronous 4-1: basal-like, 4-2: Her2-enriched. We used six months as the cut-point to define synchronous and metachronous cancers [7]. More detailed information on the four pairs of BBC is shown in Table 1. We also sequenced two pairs of primary breast cancer samples and corresponding metastatic liver samples, which served as a control group (Table 1).

**Table 1:** Clinical characteristics of patients with bilateral breast cancer and metastatic breast cancer

Patient ID	Tumor 1				Tumor 2				Diagnosis
	Age (years)	Anatomical site	Subtype	TNM stage	Time since cancer 1	Anatomical site	Subtype/histologic type	TNM stage	
1	55	Breast	Luminal	T2N0M0	1 month	Contralateral breast	Luminal	T3N0M0	Synchronous BBC
2	55	Breast	HER2-enriched	T2N1Mx	1 month	Contralateral breast	Luminal	T1N0Mx	Synchronous BBC
3	45	Breast	Basal-like	T3N0M0	2 years	Contralateral breast	Basal-like	T1N0Mx	Metachronous BBC
4	46	Breast	HER2-enriched	T2N1M0	3 years	Contralateral breast	Basal-like	T2N0Mx	Metachronous BBC
5	41	Breast	Basal-like	T3N1M0	4 years	Liver	Mucinous adenocarcinoma	/	Metastatic breast cancer
6	49	Breast	HER2-enriched	T2N2M0	6 months	Liver	Adenocarcinoma	/	Metastatic breast cancer

BBC, bilateral breast cancer.

We achieved a median of 427.8X sequence coverage (range: 335.8X∼563.6X) of targeted exonic regions, with 97.3% of loci covered at ≥10-fold. On average, 72.8 coding mutations per tumor were identified, 30.1% of which were synonymous (Supplementary Table 1). Of coding point mutations, the observed nonsynonymous:synonymous ratio of 2.33:1 (493:212) was not significantly higher than that expected by chance (right-tail proportional test, P = 0.99) [25], indicating that the majority of coding mutations do not confer a selective advantage to BBC. This is similar to the nonsynonymous:synonymous ratio reported in basal-like breast cancer [26]. The overall BBC mutation rate was comparable to that of other subtypes of breast cancer [27, 28]. *TP53* was found to be the most commonly mutated gene in BBC (50% [4 of 8] of the samples). We observed that a nonsynonymous TP53 mutation (p.R43H) occurred in both samples from Patient 6. We also observed that Patient 3 and 4 had two mutations in TP53 at different positions, respectively. Specifically, In Patient 3, we found TP53 p.R81X (stopgain mutation) mutated in tumor1 but not tumor2, whereas TP53 p.R141H was mutated in tumor2 but not tumor1. In Patient 4, TP53 p.Y88C and p.R210X mutations were found in tumor1 and tumor2, respectively. Other known cadre genes that drive breast cancer clusters (i.e., *PIK3CA*, *GATA3*, *CDH1*, and *MAP3K1* [27-30]) were not found, perhaps due to small number of samples in this study and/or heterogeneity of breast cancer.

**Figure 1:**
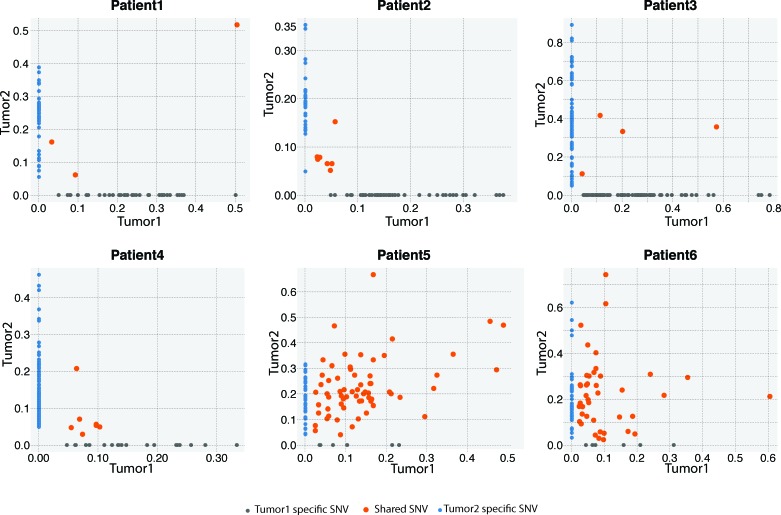
Variant frequency (VAF) distribution of identified SNVs between the first and second tumors from the six pairs of tumors in the exome-sequencing screen

To determine whether BBCs are genetically independent, we first compared the similarities of mutations between paired BBC samples and breast/metastasis samples (Figure 1). The majority of somatic mutations in primary tumors are shared with the corresponding metastatic lesions [31, 32]. In our study, concordant mutations were rare (<10%) in the paired BBC samples, in sharp contrast to around 80% concordant mutations in the paired breast/metastasis samples (median depth: 60.9X, range: 51.8X∼81.6X). We have identified 21 mutations shared in the BBC tumor 1 and tumor 2 (Supplementary Table 2). Among these, there are only two non-synonymous somatic mutations identified in Patient 4, i.e. *NBPF1* p.D896Y and *LILRA6* p. Y297S. The first mutation was predicted to exert functional impact (SIFT score = 0.01) thus considered potential driver mutation, whereas the second did not (SIFT score = 0.11). If BBCs are origin dependent, they will share more SNVs than are expected by chance. Fisher's exact test was used to determine whether two BBCs shared a significant number of somatic SNVs. The P values for the Fisher's exact test were not significant for all four pairs of synchronous or metachronous BBCs but were highly significant for the paired breast/metastasis samples (Table 2).

**Table 2:** Fisher's exact test to determine the clonality between the 1st and the 2nd tumors by evaluating the concordance of their somatic SNVs

Patient ID	Number of mutations^A^			*P* value	*FDR*
	Tumor 1	Tumor 2	Shared		
1	40	46	3	0.46	0.68
2	63	38	7	0.05	0.10
3	91	81	4	0.99	0.99
4	28	185	7	0.56	0.68
5	70	95	64	8.80E-44	5.28E-43
6	47	89	41	7.36E-29	2.21E-28

A only exonic mutations were included. 1-4, BBC patients; 5-6,metastatic breast cancer patients.

**Figure 2:**
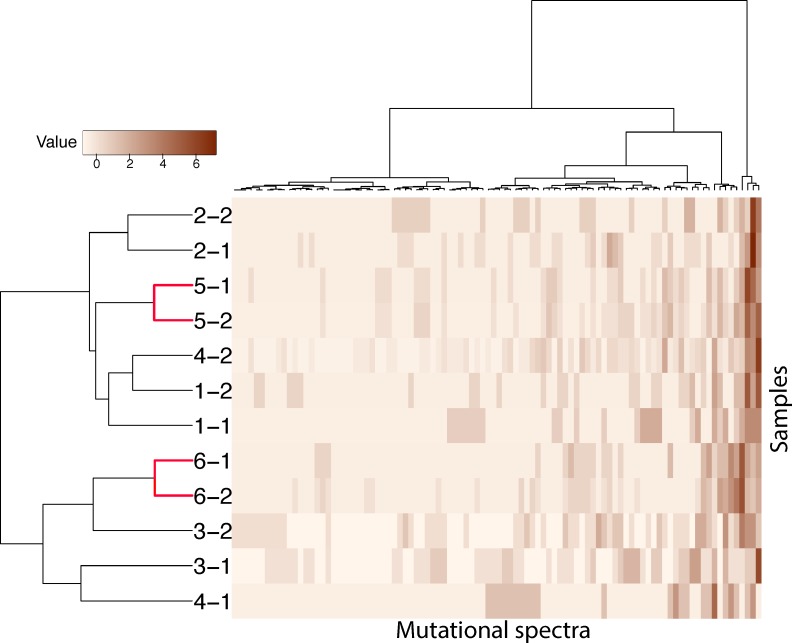
Hierarchical clustering of 12 samples according to their nucleotide context-specific, exonic, and somatic mutation rates in the exome sequencing screen Mutation spectra in each sample were scaled. Each row represents a sample, and each column represents 1 of 96 strand-collapsed trinucleotide context mutation signatures. Top bar, single-nucleotide context mutational signature; left bar, cluster membership; right gradient, mutation rate scale. “-1”: first tumor, “-2”: second tumor.

Different combinations of mutation types, termed mutational signatures, reflect different mutational processes that are operative in cancer [33]. The transition:transversion ratio in BBC was 1.5 in this study, similar to that reported in other types of breast cancer [34]. The mutational signatures of the paired samples are shown in Supplementary Figure 1, with the most common mutations of C>T nucleotide transition (C>T:G>A) in all cases [33]. Consistently, a clustering analysis based on the mutational signature of the 12 samples matched the two pairs of primary/metastasis samples; the BBCs were mismatched except in patient #2 (Figure 2).

To get another line of evidence whether BBCs are origin independent, we used aCGH to study copy number alteration landscape of all these tumors. Although there was inter-patient diversity in the copy number change per chromosome arm among all the six paired cases, the overall pattern of large copy number alterations seen in the first and second samples (Supplementary Figure 2) was consistent with that of these well described alterations. For example, we observed frequent large chromosomal gains in 1q, 3q, 8q, 20q, and 21q and broad losses involving 8p, 11q, 16q, and Xq [16-18, 35, 36]. Thus, these BBC tumor samples, as well as the paired liver samples from metastatic breast cancer, bore many of the hallmark copy number alterations commonly found in breast cancer.

With regard to the inner concordance between the two tumors from each patient, two metastatic breast cancer patients (#5 and #6) had identical CGH ratio profiles in their corresponding tumor pairs. All other BBC tumor pairs had dissimilar CGH ratio profiles (Supplementary Figure 2). More direct evidence resulted from the subsequent clonality analysis using CNV profiles (Table 3). We calculated the LR2 to evaluate the clonal relatedness of the six pairs of tumors and found a significantly higher LR2 value for the two primary-metastasis pairs (P<0.001); however, no obvious relationships were observed for the four synchronous or metachronous BBCs (P value >0.05).

**Table 3:** Clonality analysis of 12 samples according to their copy number variations at the probe level

Sample 1	Sample 2	LR2 statistic	LR2 p value
1-1	1-2	2.53E-03	0.35
2-1	2-2	1.12E-02	0.18
3-1	3-2	3.10E-03	0.33
4-1	4-2	9.90E-04	0.47
5-1	5-2	1.35E+27	0.00
6-1	6-2	2.67E+09	0.00

LR2: Likelihood ratio 2, quantifies the odds that the two tumors are clonal.

-1: First tumor, -2: second tumor.

1-4, BBC patients; 5-6, metastatic breast cancer patients.

## DISCUSSION

Breast cancer patients have a 2- to 6-fold higher risk of developing contralateral breast cancer than women in the general population have of developing a primary breast cancer [1]. BBC is presumed to have a unique genetic background, considering that BBC patients are younger, more commonly have a family history and *BRCA* germline mutations than unilateral breast cancer cases [8, 9, 37]. Only a few reports have presented the genetic findings of sporadic BBCs involving different aspects and factors in breast carcinogenesis [17].

Another dilemma in BBC is its unclear origin, as it is unknown whether it represents an independent second primary tumor or dependent on the primary tumor. Clarifying this issue will have implications for treating BBC and understanding its carcinogenesis [16, 24, 38]. The clonality of multiple malignancies has long been of interest to cancer biologists. However, the clinical and histopathologic features at the individual patient level described in previous studies, seem to have little diagnostic value in differentiating between an independent primary tumor and a breast-to-breast metastasis [14]. Recent advances in molecular biology have made it possible to define the relationship between the two BBC tumors. A comparative analysis using various cytogenetic techniques presented evidence of the pathogenetic independence of most BBC tumors [10-14, 16, 17]. A few studies have shown concordant genetic alterations in BBC [18, 23, 24], suggesting that comprehensive genome profiling approaches are needed to help us fully understand the discrepancies between contralateral breast metastases and de novo primary tumors. Another reason for these inconsistencies is that different criteria are used to define synchronous and metachronous BBCs [4].

In the present study, we included four paired-samples of BBCs—two synchronous and two metachronous—using the most widely used classification, a 6-month time span from the index tumor to the second tumor. Two paired primary breast tumors and corresponding metastatic liver lesions were used as positive controls for this synchronous and metachronous primary-metastasis model. We achieved a mutational SNV rate in BBCs that was comparable to that in sporadic unilateral breast cancer, as found on high-throughput exome-sequencing [27, 28]. They also shared an identical mutational signature with that of sporadic breast cancers, dominated by the most common mutation of C>T nucleotide transition (C>T:G>A) [33]. It is accepted that different molecular profiles from multiple neoplasms represent distinct clonal origins, while concordant data suggest a monoclonal origin [4]. We observed only a few overlapping variants in each bilateral breast tumor pair, which significantly differed from the obvious common variants shared by the primary and metastatic lesions of two metastatic breast cancers. The bilateral tumors in three of the four BBC patients segregated after hierarchical clustering of mutational signature data, indicating that they were independent. Our breast cancer cases with liver metastases showed proper clustering, allowing us to identify metastatic disease.

One way to reliably determine whether two bilateral breast tumors are two primary carcinomas or metastases of a primary neoplasm is by Fisher's exact test. It assesses whether two tumors are sharing a significant number of somatic mutations and provides a line of evidence for clonal dependence [39]. Using this guideline, a pathogenetic independence of the bilateral tumors was inferred in the four pairs of synchronous and metachronous BBCs. On the contrary, the likelihood that the shared mutational changes found in the tumors from metastatic breast cancer patients occurred by chance is extremely small. These findings are in keeping with those of previous studies; they exclude the hypothesis of metastatic spread and favor true bilaterality of BBC.

We obtained high-resolution views of all unbalanced chromosomal alterations in this series of six pairs of tumors. The overall pattern of frequently detected genetic alterations in BBC did not differ significantly from that found in previous studies of unilateral, sporadic breast cancers [35, 36]. Compared to the commonly used allelotyping approach (loss of heterozygosity analysis), an elaborate genome-wide copy number arrays (CGH array) may be more valuable for determining the independence or clonality of multiple cancerous lesions. Recently, clonality analysis, a more accurate statistical method with priority over preliminary hierarchical clustering, was developed to compare such genomic profiles [40]. This analysis revealed an overwhelmingly higher degree of clonal relatedness in the cytogenetic profiles of primary and metastatic tumors. By contrast, a nearly negligible association between BBCs was found for all four BBC pairs in our study, leaving little doubt that they are pathogenetically independent. This was in line with the findings that each BBC tumor is the result of a separate carcinogenic event [9].

In summary, with the help of whole-exome sequencing and CGH techniques, we systematically revealed the independent genomes of BBC in Han Chinese women, both synchronous and metachronous. Even the two synchronous tumors that occurred within as short as a month also have very diverse mutation profiles thus representing two independent tumors. This is a revelation at the genomic level that provides new insight into the development of bilateral breast cancer, although we cannot completely rule out the possibility of that they have the same clonal or sub-clonal origin because of our limited sample size. These findings appear to have a practical impact on the therapeutic regimen for BBC, as the clinical management of localized breast cancer is critically different from that of metastatic disease. It is essential to consider BBC as two diseases because similar systemic management for the two may not be applicable. Finally, this proof-of-principle study should be further tested by additional large and comprehensive research to provide important new insights into the biological mechanism of this uncommon disease.

## MATERIALS AND METHODS

### Patient selection and sample preparation

We searched a retrospective archive of the Tumor Biobank at Tianjin Medical University Cancer Institute and Hospital to identify all patients who had been consecutively histologically diagnosed with BBC from 2004 to 2010.

All patients had been treated by mastectomy or breast conservation therapy according to local protocols and had undergone resection of their bilateral breast tumors. Patients with metastatic cancer in the contralateral breast, as defined using Chaudary's criteria [41], and who developed distant disease between the development of the first and second primary breast carcinomas were excluded from the study. Synchronous and metachronous breast disease was distinguished by the development of a contralateral cancer within six months or more than six months after the initial tumor diagnosis, respectively [7]. Concomitantly, we also collected samples from 2 metastatic breast cancer patients with liver metastasis for whom both the primary tumor and the sequential hepatic metastasis were available in the Tumor Biobank at Tianjin Medical University Cancer Institute and Hospital (Table 1). Each patient donated 20 mL of blood that was collected into heparinized tubes. The Ethics Committee of Tianjin Cancer Institute approved the study protocol, and written informed consent was obtained from all patients, authorizing the genetic analysis of DNA from their biological samples for research purposes.

Data collected included patients' characteristics, perioperative age, tumor size, tumor grade, tumor histological type, and clinical stage for both the initial and contralateral tumors, as well as the time interval between the first and second tumors. Patients' estrogen, progesterone, and HER-2/neu receptor status was also noted by IHC or FISH if the immunohistochemical analysis score was +2 equivocal.

All specimens were snap-frozen in liquid nitrogen during surgery and immediately stored at −80°C for further study. Two pathologists independently confirmed the histopathological diagnosis, tumor grade, and tumor cell content of the hematoxylin-and-eosin-stained tumor sections. For all tumor-tumor pairs, representative fresh-frozen blocks with a tumor cell purity of more than 80% were selected, and genomic DNA was extracted from paired tissues and blood for sequencing using a standard protocol (Qiagen).

### Illumina-based whole-exome sequencing and reads alignment

Genomic DNA from the matched tumors and peripheral blood was fragmented and hybridized to commercially available capture arrays for enrichment. The exome capture procedure was performed with Agilent's SureSelect Human All Exon Kit protocol (Agilent Technologies). The resulting DNA libraries, with an average insert size of 200 bp, were sequenced using the 90-bp paired-end technology on Illumina HiSeq 2000. A real-time image analysis and base calling were performed with HiSeq Control software version 1.1.37 and Real-Time Analysis software version 1.7.45 using standard parameters, respectively. Before aligning reads to the *Homo sapiens* reference genome, we removed low-quality reads that met the following criteria: (1) reads that included sequencing adaptors; (2) reads with a ratio of ambiguous bases to read length ≥0.1; and (3) reads with more than 5 ambiguous bases. The resultant reads were aligned to reference genome hg19 using Burrows Wheeler Aligner software version 0.5.9 (bwa aln -o 1 -e 50 -m 100000 -t 4 -i 15 -q 10 -I) [42]. SAMtools was used to convert the SAM-formatted alignment results to BAM-formatted alignment files; the Genome Analysis Toolkit (GATK IndelRealigner) was used to calibrate alignment accuracy in local regions, and *Picard* was used to mark duplicates [43, 44].

### Detection of somatic SNVs and indels

MuTect was used to detect somatic SNVs from the germline SNVs in blood for both the discovery and validation cohorts. This sensitive tool detects somatic point mutations and addresses tumor impurity and heterogeneity [45]. The minimum coverage was set at 10X for both the tumor and germline genomes; the mutation allele fraction was ≥5%, and ≥5 reads supported this mutation. The somatic mutations were annotated with ANNOVAR [46]. VarScan2 [47] was used to detect somatic indels by comparing the tumor bam file with its matched blood bam file, using the following parameters: min-coverage, 10; min-coverage-blood, 10; min-coverage-tumor, 10; min-var-freq, 0.05; min-freq-for-hom, 0.75; somatic-p-value, 0.05; and min-avg-qual, 0. False-positive indels were removed through a filtering pipeline and manual inspection.

### Identification of somatic SNVs for multiple tumors in a patient

To detect sequential somatic SNVs in patients with more than 1 tumor and a shared normal control, the MuTect algorithm [45] was used. An SNV superset was compiled by concatenating all the detected somatic SNVs. We examined this SNV superset in the tumors with a FDR corrected binomial probability of 0.05, assuming a background sequencing error rate of 1%; SNVs with a FDR<10% were called. In this way, a few SNVs that were less sampled during sequencing were recovered.

### Copy number variation analysis by aCGH

The chromosome copy number of all tumor samples and their paired blood samples was assayed using an Agilent SurePrint G3 Human CGH microarray kit, 1×1 M, with an average probe spacing of 2.1 Kb (Agilent Technologies). Genomic DNA labeling and chip processing were performed according to Agilent's recommended protocols. After the hybridization step, microarray slides were washed and scanned using an Agilent Technologies DNA Microarray Scanner with Surescan High-Resolution Technology. Raw expression data, along with tif images, were extracted using Feature Extraction software.

Array CGH data were analyzed using Agilent CytoGenomics Edition version 2.7.22.0 software (Agilent Technologies, CA, USA), using the paired peripheral blood from each patient as germline reference. The QC metrics table was used to check the signal intensities and background noise. A derivative log ratio score above 0.20 was set as the cutoff criterion to exclude the poor quality of array data and the possibility of false copy number variation (CNV) calling [48]. A CNV analysis was performed using the Aberration Detection Method 2 algorithm with a sensitivity threshold of 6.0 and a minimum of three probes. The copy number profiles were visualized using the Integrative Genomics Viewer (IGV).

### Clonality analysis

For SNVs, we determined whether two tumors from the same patient shared a significantly higher than expected number of somatic mutations. Specifically, we created a 2-by-2 contingency table that represented the number of somatic mutations that were specific or shared between tumors from the same patients. Fisher's exact test was used to determine whether they shared a significantly higher number of somatic mutations than would be expected by chance; this was followed by the Benjamini Hochberg method of FDR control.

With respect to CNV, we used the R software package Clonality [49], which uses tumor copy number profiles at the probe level, to determine whether two tumors from the same patient were clonally or origin independent using a likelihood ratio 2 (LR2) statistic [49] (quantifying the odds that the two tumors are clonal). To run Clonality, we used DNAcopy [40] to create a copy number array object. The copy number array object was used as input for Clonality.

## SUPPLEMENTARY MATERIAL FIGURES AND TABLES


