# Supplementary material for: Comparative genomic analysis reveals bilateral breast cancers are genetically independent
Source: Oncotarget. 2015 Sep 10;6(31):31820–9. doi: 10.18632/oncotarget.5569 (PMC4741642; doi:10.18632/oncotarget.5569)
Supplement: Supplementary file 1 [file oncotarget-06-31820-s001.pdf]

Comparative genomic analysis reveals bilateral breast cancers are genetically independent

Supplementary Material

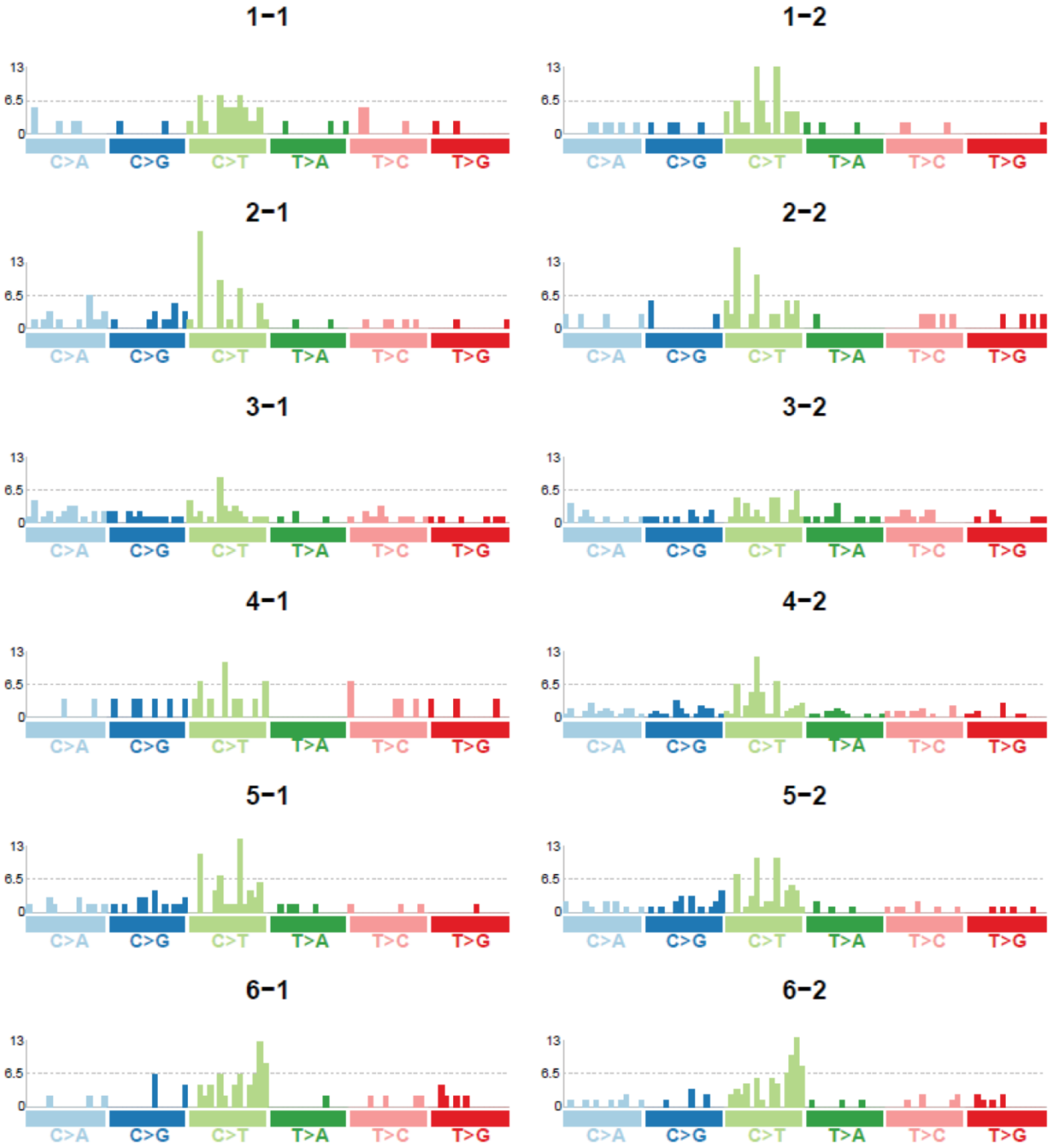

Supplementary Figure 1. Mutational signatures of the first and second tumors from the six pairs of tumors in the exome-sequencing screen. The mutational signature is displayed using a 96-substitution classification. The probability bars for each type of substitution are displayed in different colors. The mutation types are displayed on the horizontal axes, and the vertical axes represent the percentage of each type. -1: First tumor, -2: second tumor.

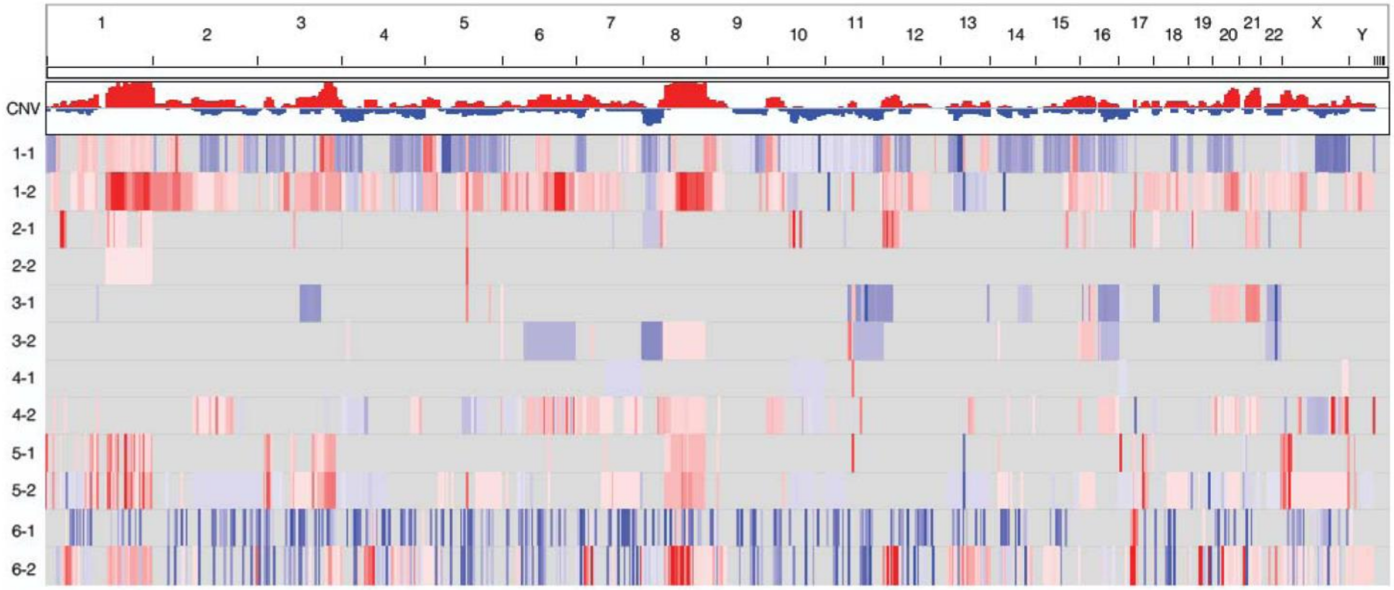

Supplementary Figure 2. Copy number variations (CNVs) for the first and second tumors from the six pairs of tumors. Copy number profiles were derived from the Agilent array data and visualized using the Integrative Genomics Viewer (IGV), with red indicating copy number gain and blue representing copy number loss. Each row represents a sample. -1: First tumor, -2: second tumor.

Supplementary Table 1. Somatic SNVs of exome sequencing data from the first and second tumors of the six pairs of breast cancer tumors[A]

Patient ID	Sample[B]	Exonic	Exonic/splicing	ncRNA exonic	ncRNA 3'UTR	Splicing	3'UTR	5'UTR	Synonymous	Nonsynonymous	Stop gain	Transversion	Transition	Total
1	1-1	30	2	1	1	0	4	2	7	25	0	12	28	40
	1-2	37	1	3	0	0	2	3	14	21	3	14	32	46
2	2-1	49	0	2	0	0	7	5	16	28	5	29	34	63
	2-2	27	0	1	0	2	5	3	8	16	3	12	26	38
3	3-1	62	1	5	0	3	10	10	16	45	2	48	43	91
	3-2	56	1	5	0	3	10	6	17	39	1	39	42	81
4	4-1	13	0	3	0	2	6	4	5	8	0	11	17	28
	4-2	149	3	3	0	3	19	8	51	96	5	79	106	185
5	5-1	59	3	1	0	0	3	4	21	39	2	28	42	70
	5-2	83	3	2	0	0	3	4	26	54	6	37	58	95
6	6-1	44	0	1	0	0	2	0	10	34	0	14	33	47
	6-2	82	0	2	0	1	3	1	21	60	1	23	66	89

[A]Based on the hg19 UCSC release of the human genome.

[B]"-1": the first tumor, "-2": the second tumor.

Supplementary Table 2. All the shared somatic mutations identified in BBCs

Chr	Start	End	Ref	Obs	Function	Gene	ExonicFunc	AAChange	Patient ID	SIFT	PolyPhen2	LJB_PhyloP	LJB_MutationTaster	LJB_LRT
chr4	88536901	88536901	C	T	exonic	DSPP	synonymous SNV	NM_014208:c.C3087T:p.N1029N	Patient 1					
chr8	86572500	86572500	G	A	UTR3	REXO1L1			Patient 1					
chr20	29637986	29637986	T	A	ncRNA_exonic	MLLT10P1			Patient 1					
chr6	136582113	136582113	A	C	UTR3	BCLAF1			Patient 2					
chr8	144732026	144732026	G	A	UTR5	ZNF623			Patient 2					
chr19	45395714	45395714	T	C	exonic	TOMM40	synonymous SNV	NM_001128917:c.T339C:p.F113F	Patient 2					
chr20	58476774	58476774	G	A	exonic	SYCP2	synonymous SNV	NM_014258:c.C1125T:p.D375D	Patient 2					
chrX	54476149	54476149	A	G	exonic	FGD1	synonymous SNV	NM_004463:c.T2091C:p.T697T	Patient 2					
chr10	21806056	21806056	G	A	exonic	C10orf140	synonymous SNV	NM_207371:c.C696T:p.A232A	Patient 2					
chr22	28194933	28194933	T	C	exonic	MN1	synonymous SNV	NM_002430:c.A1599G:p.Q533Q	Patient 2					
chr4	9783905	9783905	C	T	exonic	DRD5	synonymous SNV	NM_000798:c.C252T:p.A84A	Patient 3					
chr7	116502563	116502563	C	G	splicing	CAPZA2			Patient 3					
chr10	135054030	135054030	A	G	UTR3	VENTX			Patient 3					
chr12	11420475	11420475	A	G	exonic	PRB3	synonymous SNV	NM_006249:c.T708C:p.P236P	Patient 3					
chr1	16893827	16893827	C	A	exonic	NBPF1	nonsynonymous SNV	NM_017940:c.G2686T:p.D896Y	Patient 4	0.01				
chr1	32661594	32661594	C	T	UTR3	TXLNA			Patient 4					
chr3	160219839	160219839	T	C	UTR3	KPNA4			Patient 4					
chr8	86572433	86572433	T	C	UTR3	REXO1L1			Patient 4					
chr19	54744772	54744772	T	G	exonic	LILRA6	nonsynonymous SNV	NM_024318:c.A890C:p.Y297S	Patient 4	0.11	0.450564	0.980991	0.059101	0.97129
chr19	54744777	54744777	C	T	exonic	LILRA6	synonymous SNV	NM_024318:c.G885A:p.R295R	Patient 4					
chr1	159896889	159896889	G	C	UTR3	IGSF9			Patient 4					

Notes: SIFT, Polyphen2, LJB_Phylop, LJB_MutationTaster and LJB_LRT are different metrics of predicted functional impact via different strategies. Generally speaking, SIFT < 0.05 is considered that this mutation exerts functional impact; whereas mutation is considered damaging if Polyphen2, LJB_Phylop, LJB_MutationTaster or LJB_LRT > 0.85.
